# Cancer heterogeneity: converting a limitation into a source of biologic information

**DOI:** 10.1186/s12967-017-1290-9

**Published:** 2017-09-08

**Authors:** Albert Rübben, Arturo Araujo

**Affiliations:** 1 Department of Dermatology, Euregio Skin Cancer Center, University Hospital of the RWTH Aachen, Aachen, Germany; 2 Integrated Mathematical Oncology Department, Moffitt Cancer Center and Research Institute, Tampa, FL USA

**Keywords:** Somatic cancer evolution, Genetic heterogeneity, Parallel evolution, Punctuated evolution

## Abstract

Analysis of spatial and temporal genetic heterogeneity in human cancers has revealed that somatic cancer evolution in most cancers is not a simple linear process composed of a few sequential steps of mutation acquisitions and clonal expansions. Parallel evolution has been observed in many early human cancers resulting in genetic heterogeneity as well as multilineage progression. Moreover, aneuploidy as well as structural chromosomal aberrations seems to be acquired in a non-linear, punctuated mode where most aberrations occur at early stages of somatic cancer evolution. At later stages, the cancer genomes seem to get stabilized and acquire only few additional rearrangements. While parallel evolution suggests positive selection of driver mutations at early stages of somatic cancer evolution, stabilization of structural aberrations at later stages suggests that negative selection takes effect when cancer cells progressively lose their tolerance towards additional mutation acquisition. Mixing of genetically heterogeneous subclones in cancer samples reduces sensitivity of mutation detection. Moreover, driver mutations present only in a fraction of cancer cells are more likely to be mistaken for passenger mutations. Therefore, genetic heterogeneity may be considered a limitation negatively affecting detection sensitivity of driver mutations. On the other hand, identification of subclones and subclone lineages in human cancers may lead to a more profound understanding of the selective forces which shape somatic cancer evolution in human cancers. Identification of parallel evolution by analyzing spatial heterogeneity may hint to driver mutations which might represent additional therapeutic targets besides driver mutations present in a monoclonal state. Likewise, stabilization of cancer genomes which can be identified by analyzing temporal genetic heterogeneity might hint to genes and pathways which have become essential for survival of cancer cell lineages at later stages of cancer evolution. These genes and pathways might also constitute patient specific therapeutic targets.

## Background

Malignant tumors can display a high degree of spatial and temporal genetic heterogeneity [1, 2]. Genetic heterogeneity is the result of multilineage somatic evolution of genetically unstable cancer cells and it is regarded as the main reason for failure of classic cytotoxic drugs, as well as modern targeted therapy [2]. In this review, we would like to present evidence for the assumption that analyzing spatial and temporal genetic heterogeneity enhances the information content of molecular cancer profiling, key to identifying suitable patient-specific therapeutic targets.

## Characteristics of human cancer evolution

### Mutation load does not increase linearly with cancer progression

The identification of only a few and specific mutations in colon cancer has suggested that malignant progression proceeds with the continuous accumulation of a limited number of oncogenic mutations followed by clonal expansion of the mutated subclones resulting in a linear multistep process of cancer evolution [3]. Subsequent research on somatic cancer evolution which analyzed primary tumors and metastases in individual patients [4, 5], complex chromosome rearrangement events in single cancers [1, 6–8] or multiple single cells within one malignant tumor [1, 2, 9–11] could not confirm this assumption. In contrast, most data suggest a non-linear accumulation of structural and numeric chromosomal aberrations as well as of gene mutations. This has been denominated punctuated evolution [10].

Chromosome aberrations such as aneuploidy, more complex chromosomal rearrangement as well as abundant gene copy number variations can be found in early stages of malignant progression and these structural mutations seem to get stabilized at later stages of somatic cancer evolution and clinical progression. This has been demonstrated in many tumors such as malignant melanoma, breast cancer, pancreatic cancer and prostate cancer [1, 2, 4–11].

Point mutations seem to be acquired more steadily but at least in melanoma, more advanced tumors do not always display higher mutation loads [12, 13].

This insight into somatic cancer evolution raises two major questions:

- Why do cancer cells acquire most structural mutations (aneuploidy, rearrangements, translocations, gene copy number variations) and probably most driving point mutations during early stages of carcinogenesis where molecular and histologic signs of genome destabilization such as atypical mitoses are less apparent compared to metastasized cancer cells?
- What are the mechanisms responsible for the apparent stabilization of the cancer genomes at later stages of malignant progression?

### Aneuploidy is pseudo-stabilized under constant selective pressure

Aneuploidy has fascinated generations of pathologists as atypical mitoses, a key process leading to unequal distribution of chromosomes are visible under the microscope in cancer samples [14]. In addition, aneuploidy may be directly visible as the presence of gigantic nuclei, of multinucleate cells as well as of cancer cell nuclei which strongly vary in size. There has been a long debate on whether aneuploidy is one driver or the only driver of carcinogenesis [15] but most scientists would agree that under the concept of carcinogenesis and cancer progression as an evolutionary process, aneuploidy represents one form of genetic instability which accelerates somatic cancer evolution. An inherent feature of aneuploidy is that one missegregation event during a cancer cell division results in the duplication or loss of thousands of genes which profoundly alters the genetic composition of the progenitor cells [15–17]. It has been speculated that this feature of aneuploidy allows cancer cells to respond much faster towards environmental changes than by acquisition of point mutations. It has to be mentioned that aneuploidy is not the only mechanism leading to multiple gene dosage changes through one rearrangement event. The same effect will be achieved by other complex structural rearrangements of chromosomes or chromosome parts such as chromotripsis and chromoplexy [5, 18, 19]. It might be expected that aneuploid cancer cells constantly change their chromosome composition, but the genomes of aneuploidy cancers remain remarkably stable at later stages of cancer progression [11]. This behavior of aneuploidy cells has been replicated in cell culture. It could be demonstrated that the chromosome composition of aneuploidy cancer cell lines is not by itself stable, but that it may oscillate for many passages around a predominant karyotype [16].

### Parallel (convergent) somatic evolution is frequent in human cancers

The analysis of subclone fate during somatic cancer evolution in different cancers could demonstrate that subclones may evolve through independent mutations but targetting the same genes, chromosome regions or the same molecular pathways [4, 5, 20–25]. This parallel and multilineage somatic cancer evolution has been explained by convergent phenotypic tumor evolution [20]. It has been proposed to use the term “parallel evolution” to describe these kinds of same-target independent mutations with one monoclonal origin and the term “convergent evolution” to describe parallel mutation acquisition in subclones originating from separate cancer initiating cells [24]. Parallel tumor evolution indicates a strong selective pressure for additional mutations on an already clonally expanded cell clone. In several studies it has been shown that parallel tumor evolution targets classic oncogenes as well as tumor suppressor genes [4, 22–25]. This suggests that selective pressure for cell autonomy and proliferative potential plays an important role during this phase of cancer evolution.

### Non-linearity of somatic cancer evolution can be explained by a shift from external selective pressure to internal cancer genome-mediated selective pressure

A continuous accumulation of gene mutations and structural mutations within a cancer subclone lineage should be expected under the assumption that carcinogenesis as well as cancer progression represents an evolutionary process. In the absence of strong selection, mutations loads in progenitor cells should increase proportionally depending on the mutation rate. As fidelity of DNA replication as well as fidelity of equal chromosome distribution during mitosis should decrease in cancer cells with increasing damage of genes implicated in DNA repair, DNA replication and mitosis, the mutation rates of both structural mutations and gene mutations should increase during cancer progression [17]. Therefore, one would expect that most gene mutations as well as structural mutations occur at later stages of cancer progression. As this assumption could not be validated, the premise of absent or weak selection must be wrong.

In most cancers a specific mutation may be detected which is present in all cancer cells and thereby defines the monoclonal origin of the cancer. Often, this mutation is cell type specific, targets an oncogene or inactivates both copies of a tumor suppressor gene, induces proliferation and thus leads to an initial clonal expansion. This initial and positive selection is mediated by the microenvironment and the differentiation status of the cell which together allow that a specific mutation results in cell proliferation. In most cases this expanded cell clone must accumulate further mutations leading to sequential or parallel subclone formation for full malignant conversion. Parallel evolution in this second stage indicates that selection indeed plays a significant role in cancer evolution.

In Darwinian evolution, mutation acquisition in subclones creates subclone heterogeneity which is then reduced by positive selection of the fittest subclones and negative selection of clones with lower fitness or lethal mutations. Detectable genetic heterogeneity as well as mutation load is therefore modified by the selective pressure. The high degree of genetic heterogeneity, the already significant mutation load in early stage cancers as well as the selection of mutated genes suggest that the initial clonal expansions are positively selected by the requirements of the microenvironment and by the advantages of genetic instability. In the majority of all human cancers, genetic instability is present in the form of chromosomal instability; a minority of cancers evolves through genetic instability at the nucleotide level. Genetic instability can be detected already in early cancers and even in cancer precursors. At later stages, both forms of genetic instability may be present simultaneously in cancer cells or subclones [4]. The early appearance of genetic instability can be explained by the observation that most cancers require multiple mutations for full malignant transformation. Only subclones with defects which reduce genetic stability will acquire the necessary set of mutations during the short life time of the organism and the limited proliferation potential of precancerous cells.

Early selection for chromosomal instability will lead to early acquisition of structural aberrations which confer a fitness advantage. Additional structural aberrations may occur when proliferation approaches the Hayflick limit and shortening of telomeres impedes regular chromosome distribution during mitosis. Overcoming the Hayflick limit can lead to telomere-driven chromosomal instability and most probably represents an additional non-linear event leading to profoundly rearranged cancer genomes.

As it is very unlikely that genetically unstable cancer cells stabilize their genomes at later stages through uprated mechanisms of gene and chromosome repair, one must hypothesize that with accumulating mutation load in genes and increasing chromosomal aberrations, cancer cells progressively lose their tolerance towards additional mutation acquisition. Negative selection of additional mutations is sufficient to explain the observed stabilization of cancer genomes at later stages of cancer progression within an evolutionary context. Rasnick has postulated in 2002 that there exists a point of maximum disorder of the genome that still sustains life [26]. A similar hypothesis states that restrictive effects of the genome architecture on lineage selection during somatic cancer evolution are mediated by the reduction of functional genome redundancy due to significant loss of genetic material by chromosomal instability and progressive inactivation of genes by crippling mutations [27]. Computer simulations of chromosomally unstable genomes further support the assumption of negative constraints on cancer evolution mediated by a rearranged cancer genome [27, 28]. A recent study on methylation patterns has suggested that epigenomic reprogramming rather than additional mutations might be responsible for phenotypic heterogeneity of cancer subclones at later stages of somatic cancer evolution [29]. Analysis of resistance mechanisms to immunotherapy of malignant melanoma also suggest that changes in gene expression not directly related to specific gene mutations might enhance phenotypic variability [30]. Epigenomic reprogramming might therefore constitute an alternative or additional mechanism responsible for apparent stabilization of cancer genomes at later stages of cancer progression.

Not all chromosomal rearrangements will result in negative constraints. Doubling of genes and especially whole genome doubling leading to tetraploidy seems to relieve negative constraints of chromosomal instability [31]. Genome doublings can be detected in all stages of cancer progression. Early events may induce chromosomal instability and thereby accelerate carcinogenesis while late events may enhance viability of cancer cell population by enhancing tolerance to chromosomal instability and gene loss [31]. This interpretation is in line with the hypothesis of restrictive effects of the genome architecture on lineage selection as gene doublings increase functional genome redundancy.

**Fig. 1:**
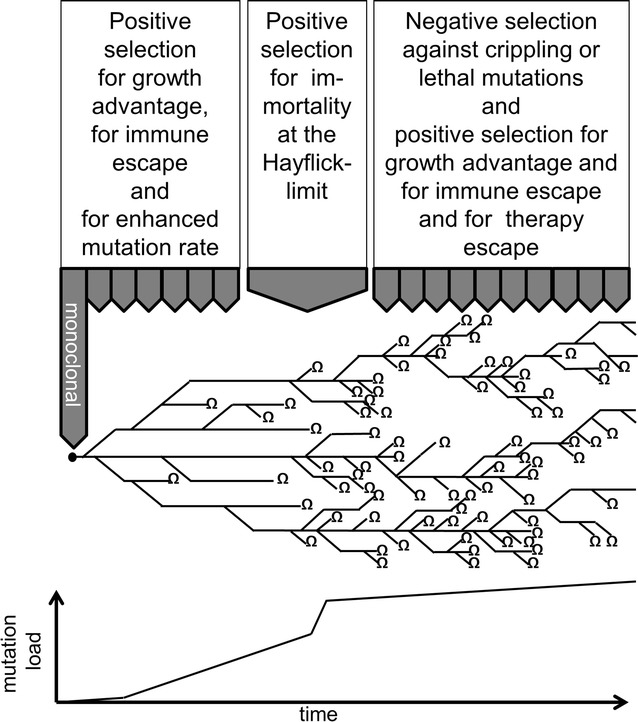
Schematic representation of somatic cancer evolution as a phylogenetic tree with early parallel evolution and strong negative selection at later stages of cancer progression. This results in a strong early rise of mutation load and structural rearrangements followed by limited further increase of mutation load after cancer cells have reached a point of maximum tolerable disorder of the genome. *Ω* indicates extinction of a subclone lineage due to lethal mutations

Figure 1 describes the concept that early carcinogenesis and somatic cancer evolution is driven primarily by positive external selective pressures on the background of genetic instability which leads to genetic heterogeneity while negative selection due to constraints of the cancer genome prevails during later cancer evolution and thereby stabilizes the initial chromosomal rearrangements detected in subclone lineages. A deduction of this concept is that analyzing different subclones during early cancer evolution reveals which mutations, genes and pathways are positively selected in the cancer while analyzing subclones during late cancer evolution will reveal which genes and pathways have become essential for survival of progressed cancer cells.

### Parallel multilineage evolution and aneuploidy reduce the detection threshold of driving mutations

During early somatic cancer evolution, oncogenic pathways may be targeted independently in different subclone lineages through individual activating mutations. In the monoclonal state, hemizygous activating mutations may suffice for clonal expansion. If two different mutations target the same pathway in two subclones of similar size, each mutation might represent less than 25% of the detected DNA sequences given the admixture with normal cells in all cancer samples. With additional subclone formation, driving mutations might be further diluted and thereby escape detection. Detection of hemizygeously or homozygeously deleted tumor suppressor genes might become even more demanding when present in only a fraction of subclones [25].

### Analysis of spatial and temporal heterogeneity allows the identification of evolutionary pathways and putative new therapeutic targets

**Fig. 2:**
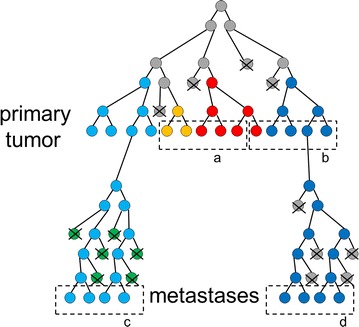
Schematic representation of somatic cancer evolution as a phylogenetic tree. *Different colors* represent subclones and indicate genetic heterogeneity in the primary tumor and its metastases. *a*, *b*, *c*, *d* indicate sampling of cancer specimens. Spatial heterogeneity is detected by sampling and analyzing either *a* and *b* or *c* and *d* and will result in an enhanced sensitivity for detection of subclones and mutations. Analyzing *a* or *b* together with *c* or *d* will reveal temporal heterogeneity. *X* indicates extinction of a subclone

Genetic analysis of multiple areas of a primary tumor will reduce admixture of subclones, if present, and thereby enhance the sensitivity of detection of driving mutations as well as detection of gene deletions (Fig. 2). It will also allow the identification of parallel evolution and thereby the identification of positively selected evolutionary pathways in cancer specimens. The knowledge of positively selected evolutionary pathways in individual patients provides two-sided information: On one hand, targeting an activated or inactivated signal transduction pathway which is positively selected in identified cancer subclones might also prove effective in not yet identified cancer subclones of the same patient. On the other hand, identification of parallel evolution with strong selection of an activated or inactivated signal transduction pathway might indicate that different subclones harboring mutations in different genes of the same pathway might already exist in the patient which reduces clinical efficacy of therapeutic targeting of the predominant mutation.

If one assumes the premise that the cancer genome is stabilized at later stages of cancer progression by negative selection, then the genetic analysis of multiple areas of one metastasis will probably not provide additional information. In contrast, analysis of different metastases in one patient will allow distinguishing individual subclonal lineages as a metastasis should represent a monoclonal proliferation originating from one single cell of one lineage (Fig. 2).

Analysis of the temporal heterogeneity during somatic cancer progression by sampling the primary tumor and its metastases or by analyzing metastases before and after therapy will also allow distinguishing individual subclone lineages (Fig. 2). Furthermore, this approach might identify changes within the cancer genomes which represent adaptations to the selective pressure induced by treatment and it might reveal which mutations, non-mutated genes and pathways have been conserved during somatic cancer evolution despite ongoing mutation acquisition. Both, mutations which evolve under treatment and genes and pathways which are conserved during progression might represent additional therapeutic targets besides known driving mutations.

## The amount of biological information deduced from analyzing genetic heterogeneity depends on the applied molecular techniques

The amount of biological information which can be deduced from analyzing genetic heterogeneity in cancer samples depends on the applied molecular techniques. Used methods for molecular profiling vary significantly with regard to their spatial resolution, the amount of parallel information they are able to deliver and their requirement for input tissue. Accordingly, the gain in biological information obtained by analyzing multiple cancer specimens or multiple single cells also depends on the used technique (Table 1).

**Table 1:** Additional biologic information through analysis of genetic heterogeneity

Technique	Spatial heterogeneity within the primary tumor or within metastases	Temporal heterogeneity during cancer progression
SNP-array, CGH, LOH-microsatellite analysis	Lower detection threshold for structural chromosomal aberrations and tumor suppressor gene deletions In the primary tumor: identification and differentiation of subclone lineages and of parallel evolution In metastases: identification of conserved genes	Lower detection threshold for structural chromosomal aberrations and tumor suppressor gene deletions Identification and differentiation of subclone lineages Identification of positive and negative selective pressure for the presence or loss of genes and pathways Identification of adaption mechanisms of the cancer genome under treatment
Gene specific Sanger-sequencing	Lower detection threshold for identification of driving mutations in the primary tumor: identification of subclones and of parallel evolution	Lower detection threshold for identification of driving mutations Identification of subclones Identification of positive selective pressure for gene mutations Identification of adaptive mutations under treatment
NGS on pooled cells	Lower detection threshold for identification of driving mutations Lower detection threshold for structural chromosomal aberrations and tumor suppressor gene deletions In the primary tumor: identification and differentiation of subclone lineages In metastases: identification of conserved genes and pathways	In addition: identification of adaptive mutations under treatment
NGS on single cells	Highest detection sensitivity for driving mutations, tumor suppressor gene deletions and structural chromosomal aberrations Identification and differentiation of subclone lineages Identification of conserved genes and pathways	In addition: lower detection threshold for identification of driving mutations Identification of adaptive mutations under treatment
Sanger sequencing or NGS of liquid biopsy	–	Identification and differentiation of subclone lineages Identification of adaptive mutations under treatment

Comparative genomic hybridization (CGH) will detect imbalances of genetic content which resulted from aneuploidy or from complex structural rearrangements. As CGH covers the whole genome, it is able to provide information on the presence, amplification or loss of genetic material at multiple chromosomal loci [32]. This parallel information allows distinguishing subclones as well as identification of conserved structural mutations during cancer progression when multiple cancer samples of one patient are analyzed, but CGH is not able to differentiate which of the two autosome chromosomes is lost or amplified in aneuploidy cancer cells. Parallel evolution of aneuploid cancer cells may involve independent loss or amplification of either autosome which may not be resolved by CGH [4]. Thus, the exact nature and the sequence of structural rearrangements during somatic cancer cell evolution may be difficult to determine by CGH alone. Moreover, detected losses or amplifications must span large regions of the genome in order to be detected and CGH may produce false positive results when the amount of input DNA falls below a threshold.

Single nucleotide polymorphism (SNP)-arrays and loss-of-heterozygosity (LOH)-microsatellite analysis represent techniques which enable parallel quantification of gene copy number alterations at multiple genetic loci but also permit assigning alleles to autologous chromosomes. Both techniques should therefore allow a more sensitive detection of parallel evolution and a better resolution of the underlying structural rearrangements compared to CGH. SNP-arrays and LOH analysis still require significant amounts of input DNA when multiple genetic regions are analyzed.

Single cell analysis of structural rearrangements can be achieved by fluorescence in situ hybridization (FISH), but this technique provides information on only a limited number of genetic loci which reduces its ability to differentiate between multiple subclones.

Detection of gene mutations by Sanger sequencing has a high sensitivity for identifying driver mutations in oncogenes or inactivating mutations in tumor suppressor genes. Sanger sequencing may be performed on single cells but the amount of input DNA rises with the number of analyzed genes. Subclones can be identified by individual mutations only when multiple areas of a primary tumor or the primary tumor and its metastases are analyzed. Subclones with the same set of driving mutations but with different chromosomal rearrangements may not be distinguished by Sanger sequencing alone. As cancers contain only few driving mutations despite a high overall mutation load, Sanger sequencing of oncogenes or tumor suppressor genes will only provide a limited amount of information on genetic heterogeneity compared to CGH or SNP-arrays and it does not confer information on structural rearrangements which is necessary to identify conserved structural mutations or conserved essential genes during cancer progression.

As each technique has its specific limitations, combination of different techniques has been advocated for reconstruction of somatic cancer cell evolution and detection of genetic heterogeneity but this approach further enhances the amount of input DNA which in turn reduces its spatial resolution [27].

A breakthrough in analyzing genetic heterogeneity as well as structural rearrangements has been provided by next generation sequencing (NGS) [1, 2, 5, 6]. When NGS is used on selected genes, multiple genes may be analyzed in parallel using very little input DNA providing resolution of genetic heterogeneity even at the single cell level. The most significant information gain is provided when NGS involves whole genome sequencing as this approach further allows detection of deletions, insertions as well as chromosome breaks and fusions. These can be used to detect complex chromosomal rearrangements as well as to reconstruct the phylogeny of structural rearrangements during somatic cancer evolution [33, 34]. Thereby, whole exome or whole genome sequencing should be able to identify conserved mutations, conserved non-mutated genes and conserved pathways during somatic cancer evolution which might represent additional therapeutic targets.

Mutations in cancers may also be detected in the serum of the patients (=liquid biopsy) as tumors release their DNA by necrosis or apoptosis to the blood stream in the form of cell free DNA. Although DNA detected by liquid biopsy should represent a mixture of subclones, sampling of cell free DNA at different time points and before and after treatment should enable the identification of subclones as well as of adaptive mutations conferring resistance to treatment [35].

Genetic heterogeneity, has been made responsible for primary and secondary resistance to classic as well as targeted therapies [36]. Whether resistant cell clones are present already before therapy or are evolving under therapy-induced selective pressure is difficult to differentiate as the resistant cell clone might represent only a small fraction of the whole tumor mass and might thereby escape molecular detection. An important conceptual advance is the assumption that early parallel evolution of driver mutations is likely to confer primary resistance to treatment and that early parallel evolution is more likely to occur in cancers which are positively selected by mutations providing only a small increase in fitness. In contrast, mutations conferring a strong increase in fitness will place the cancer cell lineage on a fitness peak and thereby promote linear evolution and selection of resistant subclones only under therapy [36]. Resistance mechanisms have been studied extensively in BRAF-mutated melanomas and it could be demonstrated that various genetic mechanisms ranging from BRAF gene amplification to alternative driver gene mutations may mediate BRAF inhibitor resistance [37]. Molecular mechanisms of resistance to novel immunotherapies with checkpoint-inhibitors have also been identified in malignant melanoma [30]. Nevertheless, analysis of genetic heterogeneity has not entered clinical routine practice yet, mainly due to the lack of controlled studies on the impact on clinical outcome of tumor heterogeneity of specific mutations.

**Fig. 3:**
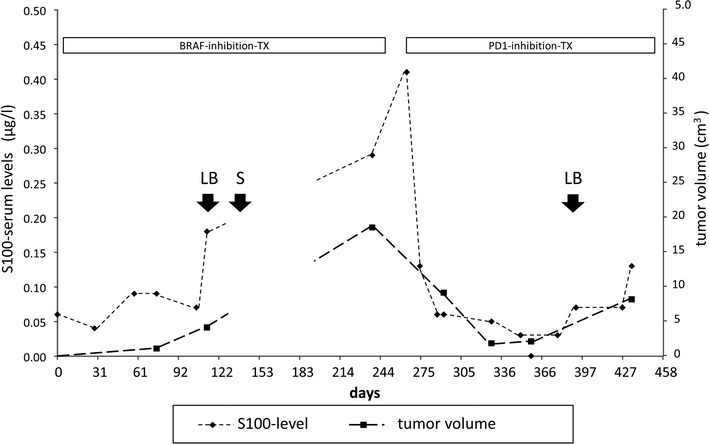
Temporal course of serum tumor marker levels and radiologic tumor mass measuring in a BRAF V600E-mutated malignant melanoma during targeted therapy with a BRAF-inhibitor and a PD1-checkpoint-inhibitor (90% PDL1-positivity of tumor cells). A rise in serum marker levels indicates emerging resistance to therapy and is confirmed by CT-scan measurement of metastasis volume. *S* actual surgery of recurrent tumor, *LB* potential time points for early liquid biopsy analysis of temporal genetic heterogeneity

Today, the conventional approach is to screen one cancer specimen for druggable mutations by Sanger sequencing or by NGS prior to initiation of targeted therapy and to repeat genetic analysis on a recurrent tumor sample in case of resistance. In this case, chances are elevated that de-novo mutations detected in the recurrence are responsible for resistance and might represent additional therapeutic targets. An approach which might gain importance in the future is the monitoring of tumor protein release to the serum during targeted or immune therapy and performing genetic analysis of cell free DNA as a liquid biopsy whenever a rise in serum tumor markers indicates enhanced tumor proliferation due to resistance to therapy (Fig. 3). This approach is based on the assumption that cycling malignant tumors constantly lose tumor proteins and tumor DNA to the serum due to significant cell death by apoptosis or necrosis. The cell loss factor of malignant tumors is estimated to range between 50% and over 90% making release of cancer proteins or DNA to the serum a valid surrogate marker for in vivo cancer cell proliferation [35, 38]. Repeated genetic analysis during therapy by liquid biopsy would not only allow correlating genetic changes to emerging resistance but would also permit identifying the subclonal composition of the treated cancer.

## Conclusions

Non-linearity of mutation acquisition during somatic cancer evolution as well as parallel evolution of cancer subclones have been described in many human cancers and they have been designated with different adjectives. Some authors focused on the primary role of aneuploidy in carcinogenesis; others have emphasized the complexity of chromosomal structural rearrangements while some have identified restrictive or permissive effects of the cancer architecture on somatic evolution. The common denominator is that cancer genomes are shaped by selective pressures and tend to become very complex already at early stages of somatic cancer evolution. Most structural and numeric chromosomal aberrations found in early cancers tend to persist at later stages despite ongoing genetic instability. Apparent stabilization of unstable cancer genomes can be explained by negative selection.

Positive selection can be easily identified at early stages of carcinogenesis and it is reflected by identification of a limited number of driving mutations in oncogenes and tumor suppressor genes. The identification of driving mutations in cancers has led to the development of targeted therapies which have significantly enlarged the armamentarium against this deadly disease. Unfortunately, many patients still succumb to cancer despite the use of targeted therapies. Genetic heterogeneity, in part due to parallel evolution, has been identified as one culprit. It has been proposed that negative selection might play an important role at later stages of cancer progression. The overwhelming numbers of mutations found in cancers do not target classic oncogenes or tumor suppressor genes. It is tempting to assume that some, if not most of these mutations are not innocuous but mediate the putative negative selection effects at later stages of cancer progression by reducing functional genome redundancy. These mutations or the remaining non-mutated pathways could represent additional therapeutic targets. The challenge will be to identify them against a background of bystander mutations.

The leading paradigm in cancer evolution is that genetic instability results in genetic heterogeneity leading to a phenotypically diverse pool of cancer subclones upon which selection can act. Identification of selection during cancer evolution has and will continue to lead to identification of therapeutic targets. Positive or negative selection cannot be assessed directly in clinical specimen but may be deduced by the analysis of genetic heterogeneity during cancer evolution. Incorporating genetic heterogeneity in the analysis of malignant tumors should therefore greatly enhance the information content of molecular analysis of cancer genomes, and help identify patient-specific therapeutic targets.

## Abbreviations

CGH: comparative genomic hybridization
SNP: single-nucleotide polymorphism
NGS: next generation sequencing
LOH: loss of heterozygosity
